# A novel *SETD2* variant causing global development delay without overgrowth in a Chinese 3-year-old boy

**DOI:** 10.3389/fgene.2023.1153284

**Published:** 2023-03-21

**Authors:** Yuanyuan Wu, Fang Liu, Ruihua Wan, Baoquan Jiao

**Affiliations:** ^1^ Department of Reproduction and Genetics, Bethune International Peace Hospital, Shijiazhuang, China; ^2^ Department of Pediatrics, Bethune International Peace Hospital, Shijiazhuang, China

**Keywords:** Luscan-Lumish syndrome, *SETD2*, overgrowth, intellectual disability, developmental delay

## Abstract

**Background:** Luscan-Lumish syndrome is characterized by macrocephaly, postnatal overgrowth, intellectual disability (ID), developmental delay (DD), which is caused by heterozygous *SETD2* (SET domain containing 2) mutations. The incidence of Luscan-Lumish syndrome is unclear. The study was conducted to provide a novel pathogenic *SETD2* variant causing atypical Luscan-Lumish syndrome and review all the published *SETD2* mutations and corresponding symptoms, comprehensively understanding the phenotypes and genotypes of *SETD2* mutations.

**Methods:** Peripheral blood samples of the proband and his parents were collected for next-generation sequencing including whole-exome sequencing (WES), copy number variation (CNV) detection and mitochondrial DNA sequencing. Identified variant was verified by Sanger sequencing. Conservative analysis and structural analysis were performed to investigate the effect of mutation. Public databases such as PubMed, Clinvar and Human Gene Mutation Database (HGMD) were used to collect all cases with *SETD2* mutations.

**Results:** A novel pathogenic *SETD2* variant (c.5835_c.5836insAGAA, p. A1946Rfs*2) was identified in a Chinese 3-year-old boy, who had speech and motor delay without overgrowth. Conservative analysis and structural analysis showed that the novel pathogenic variant would loss the conserved domains in the C-terminal region and result in loss of function of SETD2 protein. Frameshift mutations and non-sense mutations account for 68.5% of the total 51 *SETD2* point mutations, suggesting that Luscan-Lumish syndrome is likely due to loss of function of SETD2. But we failed to find an association between genotype and phenotype of *SETD2* mutations.

**Conclusion:** Our findings expand the genotype-phenotype knowledge of *SETD2-*associated neurological disorder and provide new evidence for further genetic counselling.

## 1 Introduction

Luscan-Lumish syndrome (LLS, #616831) is characterized by macrocephaly, postnatal overgrowth, obesity, intellectual disability (ID) and developmental delay (DD) (Luscan et al., 2014; Lumish et al., 2015). More variable features include Chiari malformation, advanced carpal ossification, seizures, autism/autism spectrum disorders (ASD) and other behavioral problems (Lumish et al., 2015; Lelieveld et al., 2016; Chen et al., 2021). Luscan-Lumish syndrome is an autosomal dominant neurological disease caused by heterozygous *SETD2* (SET domain containing 2) mutations (Luscan et al., 2014; Lumish et al., 2015). SETD2 is a crucial methyltransferase conserved from yeast to man, methylating substrates such as histone H3 (Edmunds et al., 2008), tubulin (Andrieux and Sadoul, 2021), and signal transducer and activator of transcription 1 (STAT1) (Chen et al., 2017). SETD2 is non-redundantly responsible for all trimethylation of lysine 36 of histone H3 and physically interacts with Pol II and various hnRNPs, which plays an important role in epigenetic modification, regulation of transcription, DNA replication and DNA damage repair (Edmunds et al., 2008; Bhattacharya et al., 2021; Molenaar and van Leeuwen, 2022; Sharda and Humphrey, 2022). Dysregulation of SETD2 function has also been reported in a series of neurological disorders and cancers including ID/DD (Faundes et al., 2018), autism/ASD (D'Gama et al., 2015), renal clear cell carcinoma (Rao et al., 2021) and acute lymphocytic leukemia (Mar et al., 2014).

The incidence of Luscan-Lumish syndrome (or *SETD2*-related neurological disorder) is unclear. In 2014, Luscan first reported two heterozygous mutations in *SETD2* gene in two patients with “Sotos-like” syndrome (Luscan et al., 2014). To date, fifty-one *SETD2* germline mutations and about thirty cases with clinical symptoms have been reported (Lumish et al., 2015; Lelieveld et al., 2016; Tlemsani et al., 2016; van Rij et al., 2018; Marzin et al., 2019; Rabin et al., 2020; Chen et al., 2021). Most of them were featured as overgrowth and speech delay. Here, we report a novel pathogenic *SETD2* variant causing global development delay without overgrowth in a Chinese 3-year-old boy and review earlier reported patients. Our findings expand the phenotype and genotype spectrum of *SETD2* mutation.

## 2 Materials and methods

### 2.1 Subjects and samples

The proband was a Chinese 3-year-old boy who was hospitalized for global development delay with his unrelated parents in October 2021. The proband was assessed using neuropsychological development checklist and performed auxiliary examinations such as magnetic resonance imaging (MRI) and electroencephalogram (EEG), G-banded karyotyping test and fragile X syndrome test. Then peripheral blood samples of the proband and his parents were collected for next-generation sequencing including whole-exome sequencing (WES), copy number variation (CNV) detection and mitochondrial DNA sequencing. Written informed consents were obtained from the patients’ parents. And this project was approved by the Ethics Committee of Bethune International Peace Hospital (Approval No. 20180023 and Approval No. 2022-KY-26).

### 2.2 Next-generation sequencing

Trio-WES, trio-CNV detection and proband mitochondrial DNA sequencing were performed in Chigene Translational Medicine Research Center (Beijing, China).

#### 2.2.1 WES

Genomic DNA of the proband and his parents was extracted from the EDTA-treated peripheral blood using the Blood Genome Column Medium Extraction Kit (Kangweishiji, China) according to the manufactural instructions. The libraries were constructed by xGen Exome Research Panel v2.0(IDT, United States) which contains 429,826 probes and targets 39 Mb protein-coding region. Illumina NovaSeq 6,000 sequencer (Illumina, United States) was used to sequence more than 99% of target sequences. After filtering and alignment, variant calling was conducted using Genome Analysis Toolkit software. Variant annotation and pathogenicity prediction were processed *via* a series of databases and software, such as 1,000 genomes, Single Nucleotide Polymorphism database (dbSNP), Exome Sequencing Project (ESP), Exome Aggregation Consortium (ExAC) and genome aggregation database (gnomAD), Provean, Sorting Intolerant From Tolerant (SIFT), Polypen2, MutationTaster, M-Cap and so on. As a prioritized pathogenicity annotation to American College of Medical Genetics and Genomics (ACMG) guideline (Richards et al., 2015), Online Mendelian Inheritance in Man (OMIM), Human Gene Mutation Database (HGMD) and ClinVar databases were used as conferences of pathogenicity of every variant. Sanger sequencing was performed to validate point mutations using 3500DX Genetic Analyzer (Applied Biosystems, United States). The primers for PCR were as follow primer: forward-TTCTTCTAGTTTTGTGCCGTTGCT, reverse primer -TGA​GAA​TAC​ATC​GCG​TGC​TCA​TAC.

#### 2.2.2 CNV detecion

Genomic DNA of the proband and his parents was extracted as above. After DNA fragmentation, genomic DNA was amplified by ligation-mediated PCR (LM-PCR) for 4-6 rounds, and then were sequenced on the DNBSEQ-T7 sequencing system (MGI, China). CNVs of 100 KB and above in length were detected using Chigene independently developed software packages for CNV detection. CNV databases such as Decipher, ClinVar, OMIM and ClinGen were used as references to annotate the pathogenic classification of each screened CNV. The biological harm and related phenotypes of CNVs were assessed by annotated information and frequency database according to ACMG practice guidelines (2019 diagnostic guidelines) (Riggs et al., 2020).

#### 2.2.3 Mitochondrial DNA sequencing

Mitochondrial DNA of the proband was extracted using the mitochondrial DNA extraction kit (Baiaolaibo, China). Full-length mitochondrial DNA was amplified using PCR and then was sheared to about 200 bp fragments using Cavoris sonicator (KU, United States). The ligated DNA products were amplified by 4–6 rounds of LM-PCR and sequenced on the DNBSEQ-T7 sequencing system (MGI, China). The variants were mapped to references mutations to find matches in the MITOMAP human mitochondrial genome database (https://www.mitomap.org/). The pathogenicity of every variant was assessed according to the MITOtip.

### 2.3 Conservative analysis of SEDT2 protein

The human protein sequences containing novel missense mutations were submitted to protein BLAST (https://blast.ncbi.nlm.nih.gov/Blast.cgi) to run a homology search. Homologous protein sequences from other species (*Pan troglodytes*, *Mus muscullus*, *Rattus norvegicus*, and *Monodelphis domestica*) were retrieved. Evolutionary conservation was analyzed using MEGA7.

### 2.4 Mutation spectrum of SEDT2 protein

By the end of 28 December 2022, we searched the public database including HGMD (https://www.hgmd.cf.ac.uk/ac/index.php), Clinvar (https://www.ncbi.nlm.nih.gov/clinvar/) and pubmed (https://pubmed.ncbi.nlm.nih.gov/) using the term “SEDT2”, “SET domain containing 2” and “Luscan-Lumish syndrome.” The two-dimensional diagram of domains and mutation sites were illustrated by DOG 2.0 software.

### 2.5 Protein visualization and structural analysis

The effect of the novel mutation on the structure of SEDT2 protein was investigated using the crystal structure of human SEDT2 protein. Three-dimensional structures of proteins were downloaded from PDB database (https://www.rcsb.org/) and AlphaFold Database (https://alphafold.ebi.ac.uk/). Virtual models of SEDT2 protein mutation analysis were performed using Pymol.

## 3 Results

### 3.1 Case presentation

The proband is a Chinese Han boy and was the only child of his parents with an unremarkable family history. His mother was 29 years old, no diabetes, hypertension, fever, or medication history during her pregnancy. His father was 30 years old and the parents were unrelated. The proband was born at a gestation age of 29^+3^ weeks because of placenta previa. He was diagnosed as neonatal respiratory distress with birth weight 1500 g (50th-90th), normal height and head circumference. The newborn was in hospital for more than 50 days and leaved hospital with good condition in all aspects. But the boy slept less, usually from 11 p.m. to 7 a.m. The proband was fed with milk powder without difficulties and complementary food was not added until 10 months old. His gastrointestinal function was poor and he was allergic to lots of food including apple, banana, wheat, fish, tomatoes, watermelon and peach. The boy presented hematochezia once at 3 months old and suffered recurrent otitis and pneumonia since the age of 7 months. This situation has improved as he grows up and he only had otitis once in the last year. He also had anemia and calcium deficiency at 7 months old, but were cured soon after symptomatic treatment.

The boy demonstrated an early development delay. He started to roll over at the age of 8 months and demonstrated severe motor and speech development delay in the first year of life. At 19 months of life, the proband was referred for clinical genetic evaluation at the Bethune International Peace Hospital. The neuropsychological development checklist showed that the proband global developmental delay with normal muscular tensity of the four limbs and normal growth (body weight 10.8 kg (25th), height 84 cm (25th-50th), head circumference 47.9 cm (50th)). Neurodevelopmental assessment of the patient was Gross motor 12 points, Fine motor 13.5 points, Adaptive ability 15 points, Language 11.5 points, Social behavior 15 points, Intellectual age 13.4 months old, Developmental Quotient 68.7, Low intelligence. After a year of rehabilitation training, the neurodevelopmental assessment of the patient at the age of 29 months old was Gross motor 21 points, Fine motor 18 points, Adaptive ability 22.5 points, Language 15 points, Social behavior 15 points, Intellectual age 18.3 months old, Developmental Quotient 63.1, Low intelligence. The latest developmental assessment was conducted on 6 January 2023 when the boy was 39.1 months old. The results showed Gross motor 24 points, Fine motor 30 points, Adaptive ability 27 points, Language 18 points, Social behavior 25.5 points, Intellectual age 24.9 months old, Developmental Quotient 63.7, Low intelligence. By the time of submitting manuscript, the proband could only speak simple words and could not jump at 3 years and 4 months old. His urination and defecation were unconscious until recently.

Cranial MRI at 19 months old demonstrated no malformation, but revealed slightly longerT2 signal at the inner edge of right cerebellar dentate nucleus and high T2 signal at the bilateral mastoid and paranasal sinuses. The recent cranial MRI and brain ultrasound showed no abnormalities (3 years old) but the EEG suggested that the proband was younger than actual age. The boy had no facial malformations and epilepsy.

### 3.2 Genetic analysis

**FIGURE 1:**
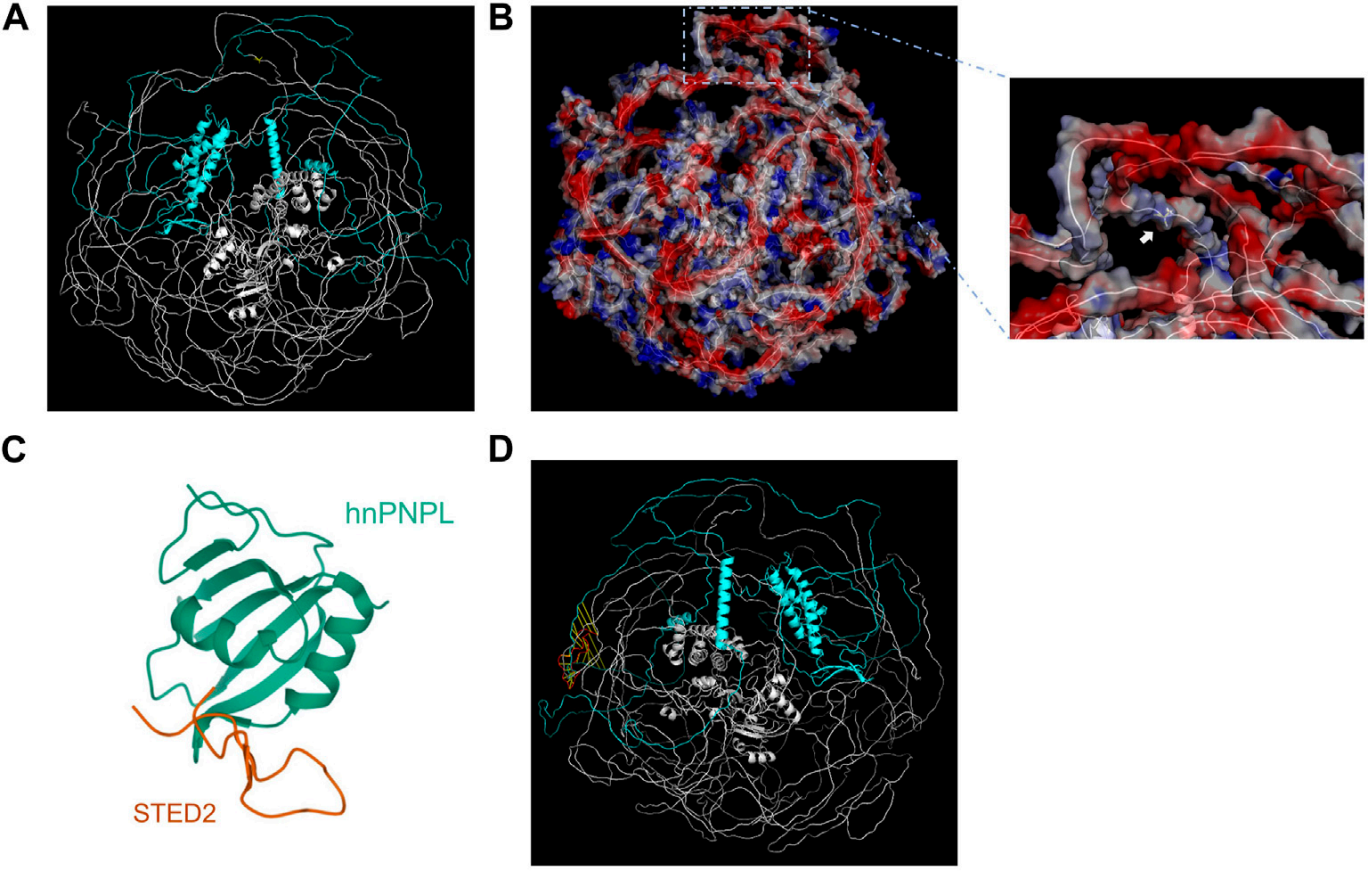
Genetic analysis of *SETD2* gene. **(A)** Pedigree diagram and Sanger sequencing result of the family. **(B)** Evolutionary conservative analysis of SETD2. 2,475–2,504 stands for the amino acid residues from the 2475th site to 2504th site located in the SRI domain of SETD2 protein. **(C)** and **(D)**. The effect of p. A1946Rfs*2 variant on the structure of SETD2 protein and overview of reported SETD2 variants.

G-banded karyotyping test and fragile X syndrome test showed no abnormalities. The high throughput sequencing was performed to detect CNV, monogenic and mitochondrial variation in the proband. By trio-WES, a novel pathogenic *SETD2* variant was identified in the proband. The boy carried a *de novo* (PS2) frameshift mutation c.5835_c.5836insAGAA (p.A1946Rfs*2, NM_014159.6) (Figure 1A), which has not been published nor reported in public databases (PM2). *SETD2* is evolutionarily conservative during various species and contains three domains in the C-terminal region (Figures 1B–D). The mutation c.5835_c.5836insAGAA would lead to the early termination of SETD2 protein and loss the SHI (SETD2-hnRNP interaction) domain (2164–2213 aa), WW domain (also known as WWP repeating motif, 2391–2422aa) and SRI (Set2-Rpb1 interaction) domain (2469–2548aa) in the wild SETD2 protein, which results in loss of function of SETD2 protein (PVS1). According to ACMG guidelines, we confirmed the variant to be pathogenic (PVS1 + PS2 + PM2). No suspicious variants were found in results of the trio-CNV detection and mitochondrial DNA sequencing.

### 3.3 Protein visualization and structural analysis

**FIGURE 2:**
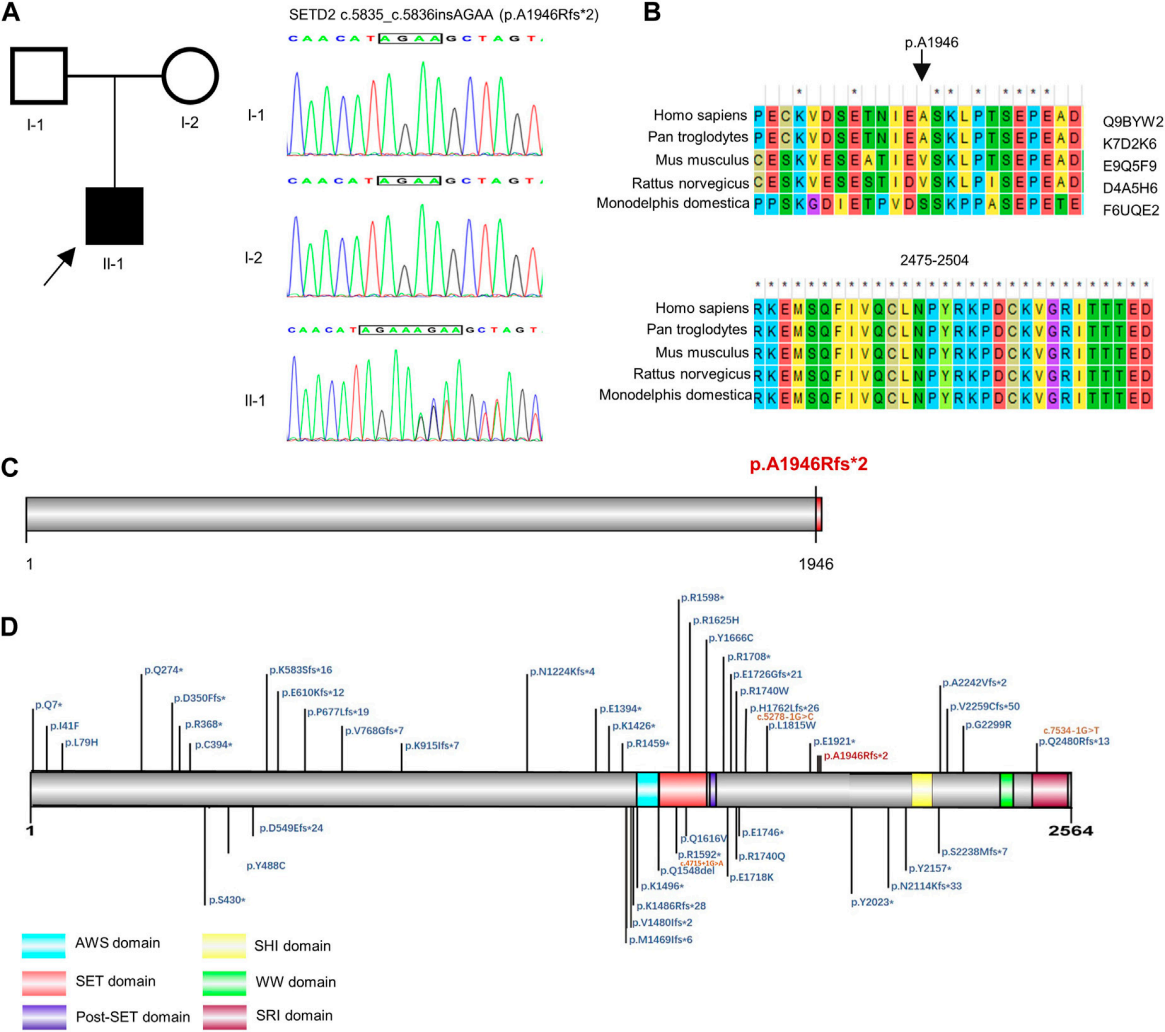
Visualization and structural analysis of SETD2 protein. The cartoon model **(A)** and surface electrostatic charge model **(B)** of SETD2 protein (AF-Q9BYW2-F1). The blue part represented the amino acid residues after the 1946th site. The yellow part represented the 1946th amino acid residue, which the white arrow pointed to. **(C)** Crystal structure of hnRNP L in complex with SETD2 (PDB:7EVR). The orange line stood for the SHI domain (2167–2192aa) from SETD2. **(D)** Alignment of SHI domains from AF-Q9BYW2-F1 and 7EVR. The red line represented the SHI domain from 7EVR.

The predicted three-dimensional structure of SETD2 was downloaded from AlphaFold Database (AF-Q9BYW2-F1) and visualized by Pymol using cartoon model and surface electrostatic charge model (Figures 2A, B). The SHI domain is located at amino acid residues after the 1946th site, which interacts with hnRNP L (Figures 2C, D). The mode of SETD2-hnRNP L interaction reveals a conserved design by which splicing regulators interact with one another. The SETD2: p. A1946Rfs*2 mutation, resulting in the lack of C-terminal region including SHI domain, may have an influence on the alternate splicing of a number of premature mRNAs.

### 3.4 Review of *SETD2* mutations and clinical symptoms

By the end of December 2022, a total of 50 *SETD2* mutationshave been reported in the public database (Supplementary Table S1). There were 18 (35.2%) frameshift mutations, 17 (33.3%) non-sense mutations, 12 (23.5%) missense mutations, 3 (6%) alternative splicing mutations and 1 (2%) in-frame deletion (Supplementary Table S1). These mutations were distributed dispersedly in the whole SETD2 region. Only 17 of them were reported with detailed clinical symptoms in 27 patients (Tables 1, 2). Including the case in this article, 92.8% (26/28) of patients showed speech and language developmental delay; 67.9% (19/28) of patients showed motor developmental delay; 66.7% (6/9) of patients presented ID; 42.3% (11/26) of patients presented macrocephaly; 16% (4/25) of patients had high stature; 29.1% (7/24) of patients were obese; 85.7% (12/14) of patients demonstrated serious behavioral problems, mainly shown as attention deficit hyperactivity disorder (ADHD), autism spectrum disorder (ASD), aggressive outbursts and anxiety disorder; 66.7% (12/18) patients experienced seizures; 81.5% (22/27) of patients had non-specific facial deformities; 70.8% (17/24) of patients manifested varying degrees of brain deformities from abnormal T2 hypersignals to classic Chiari I malformation (CIM); 66.7% (6/9) of patients had advanced bone age (Table2). In addition, some of the patients with *SETD2* mutations would present defects of multiple systems and recurrent otitis. It is worth noting that some patients bearing *SETD2* c.5218C>T mutation showed microcephaly (11/11), short stature (3/9), low weight (3/9), which were contrary to the typical features caused by other *SETD2* mutations (Table 2). The patients with c.5219G>A mutation presented normal growth (3/3) and more severe intellectual disability, which were also different from the other patients. Excluding the patients with missense mutations in the 1740th codon of *SETD2*, the rates of high stature (5/16) and macrocephaly (11/16) were higher. When comparing the symptoms including developmental delay, behavioral problems, facial and brain deformities between the different mutation types of *SETD2*, no associations between genotype and phenotype of *SETD2* mutations were found (Supplementary Table S2).

**TABLE 1:** Summary of *SETD2* gene mutations in patients with detailed symptoms.

No.	Gene	Nucleotide variant	Protein change	Category	Class	Inheritance	Diagnosis	Ref
1	*SETD2*	c.236T>A	p.L79H	missense	Pathogenic	AD/*de novo*	Luscan-Lumish syndrome	PMID:33248444
2	*SETD2* (NM_014159.6)	c.820C>T	p.Q274*	non-sense	Pathogenic	unknown	Sotos-like syndrome	PMID:24852293
3	*SETD2* (NM_014159.6)	c.1647_1667delinsAC	p.D549Efs*24	frameshift	Pathogenic	AD/*de novo*	Luscan-Lumish syndrome	PMID: 29681085
4	*SETD2*	c.2028delT	p.P677Lfs*19	frameshift	Pathogenic	AD/*de novo*	ASD and ID	PMID: 26084711
5	*SETD2* (NM_014159.6)	c.3185C>T	p.P1062L	missense	Likely Pathogenic	AD/*de novo*	ASD	PMID: 33766796
6	*SETD2* (NM_014159.6)	c.4715 + 1 G>A	—	alternative splicing	Pathogenic	AD/*de novo*	ASD	PMID: 33766796
7	*SETD2* (NM_014159.6)	c.4276A>T	p.K1426*	non-sense	Likely Pathogenic	unknown	Sotos-like syndrome	PMID: 31643139
8	*SETD2* (NM_014159.6)	c.4405dupA	p.M1469Ifs*6	frameshift	Pathogenic	AD/*de novo*	ID and macrocephaly	PMID: 27479843
9	*SETD2* (NM_014159.6)	c.4644_4646delTGC	p.Q1548del	in-frame deletion	Pathogenic	AD/*de novo*	DD and macrocephaly and tall stature	PMID: 27479843
10	*SETD2* (NM_014159.6)	c.4874G>A	p.R1625H	missense	Likely Pathogenic	unknown	Sotos-like syndrome and ID	PMID: 31643139
11	*SETD2* (NM_014159.6)	c.4997A>G	p.Y1666C	missense	Likely Pathogenic	AD/*de novo*	Sotos-like syndrome and ASD and ID	PMID: 31643139
12	*SETD2* (NM_014159.6)	c.5218C>T (12 patients)	p.R1740W	missense	Pathogenic	AD/*de novo*	microcephaly and ID and congenital anomalies	PMID: 32710489
13	*SETD2* (NM_014159.6)	c.5219G>A (3 patients)	p.R1740Q	missense	Likely Pathogenic	AD/*de novo*	ID (low normal head circumference)	PMID: 32710489
14	*SETD2*	c.5285_5286delAC	p.H1762Lfs*26	Frameshift	Pathogenic	AD/*de novo*	Sotos-like syndrome	PMID: 27317772
15	*SETD2* (NM_014159.6)	c.5835_c.5836insAGAA	p.A1946Rfs*2	Frameshift	Pathogenic	AD/*de novo*	Luscan-Lumish syndrome	this article
16	*SETD2* (NM_014159.6)	c.5444T>G	p.L1815W	missense	Pathogenic	AD/*de novo*	Sotos-like syndrome	PMID: 24852293
17	*SETD2* (NM_014159.6)	c.6471T>A	p.Y2157*	non-sense	Pathogenic	AD/*de novo*	Sotos-like syndrome and ASD and ID	PMID:31643139
18	*SETD2*	c.6775delG	p.V2259Cfs*50	frameshift	Pathogenic	AD/*de novo*	Luscan-Lumish syndrome	PMID:29681085

ASD, Autism spectrum disorders; ID, intellectual disability; DD, developmental delay.

**TABLE 2:** Summary of clinical symptoms in patients with *SETD2* gene mutation.

No.	Sex	Age (onset age)	Overgrowth			DD		ID	Behavioral issues	Seizures	Deformities		Advanced bone age	Other symptoms
			OFC	Height	Weight	Speech	Motor				Facial	Brain		
1	M	20 years (5 years)	/	+3.18 SD	+3.35 SD					/	+	CIM	/	hyperhidrosis
2	F	23 years (7 years)	>97th centile	>97th centile	>97th centile	+			attention deficit, aggressive outbursts	/	+	abnormal hypersignals around lateral ventricle	+	hypotonia; frequent ear and renal infections; polycystic ovaries
3	M	4.5 years	+3.3 SD	-	+2.5 SD	+	+	/	ASD	/	+	-	delayed bone age	recurrent otitis media; orchidopexia
4	F	17 years (6 m)	>97th centile	-	-	+	+	+	anxiety disorder, ADHD, ASD	+	/	choroid plexus cysts (prenatal); CIM & hydrocephalus	/	recurrent headaches; syringomyelia
5	F	4 years	-	-	-	+	-	/	ADHD, ASD	/	-	/	/	/
6	M	4 years	-	-	-	+	-	/	ASD, aggressive behavior	/	-	/	/	/
7	F	3 years	99.6th centile	-	-	-	-	/	attention deficit, aggressive outbursts	/	+	agenesis of the corpus callosum	/	/
8	M	11 years	+4 SD	-	-	+	-	+	/	/	+	-	/	bilateral inguinal hernia, myopia
9	M	9 years	+3 SD	+2.5 SD	-	+	+	/	aggressive	/	+	subarachnoid cyst left temporal	+	/
10	F	5.5 years	>99th centile	-	-	+	+	/	frustration intolerance, angry outbursts	/	-	bilateral ventriculomegaly	/	angioma
11	M	11 years	-	-	-	+	+	/	/	+	+	Dandy walker malformation	/	patent ductus arteriosus, bilateral cryptorchidism
12	5M	1 m-12 years	<3rd centile (11/11)	<3rd centile (3/9);	<3rd centile (3/9);	+(9/9)	+(9/9)	/	/	+(7/12)	+(12/12)	structural brain abnormalities (12/12)	/	eye abnormalities (10/12); hearing loss (8/12); genitourinary tract anomalies (11/12); cryptorchidism (5/5); congenital heart defect (11/12); hyponatremia (8/12); skeletal abnormalities;(12/12);
	7F			−(6/9)	−(6/9)									
13	2M	4.5-15 years	−(3/3)	−(3/3)	−(2/3)	+(3/3)	+(2/3)	+(2/3)	anxiety (1/3)	+(3/3)	+(2/3)	−(3/3)	+(1/3)	myopia (1/3); skeletal abnormalities;(2/3);
	1F													
14	M	12 years	+2.5 SD	+3 SD	+3 SD	+	+	+	/	/	+	/	+	hyperchromic spots, café-au-lait spots
15	F	3.3 years	-	-	-	+	+	/	-	-	-	-	/	recurrent otitis and pneumonia; food allergy
16	M	26 years	+4 SD	-	+4.5 SD	+	-	/	shyness, low sociability	/	+	/	+	/
17	M	8 years	>99th centile	>99th centile	>99th centile	+(early) -(8y)	+	+	attention deficit, ASD, aggressive outbursts	/	+	-	/	/
18	F	23 years (<3.5 years)	+2.9 SD	-	BMI32.3	+	+	/	ASD, self-mutilation, aggressive outbursts;	/	+	thickened corpus callosum	+	recurrent otitis, nasal polyps

OFC, occipitofrontal circumference; DD, developmental delay; ID, intellectual disability; M, male; F, female; +, abnormality found; -, no abnormality found; /, not mentioned; ASD, autism spectrum disorder; ADHD, attention deficit hyperactivity disorder; CIM, Chiari I malformation; BMI, body mass index.

## 4 Discussion

Overgrowth is classically defined as occipitofrontal circumference (OFC) and height>97th centile or +2SD (Moirangthem et al., 2021). Luscan-Lumish syndrome (also known as *SETD2*-related neurological disorder) is mainly characterized by macrocephaly, tall stature and ID/DD, which is similar to a series of disorders with overgrowth and learning disability containing Sotos syndrome, Mylan syndrome, Weaver syndrome, Cohen-Gibson syndrome and so forth (Pappas et al., 1993). The molecular mechanisms of these disorders primarily involve in the epigenetic modulations (like *STED2*, *NSD1*, *EZH2*), transcription factors (like *NFIA*, *NFIB*, *BRWD3*) and PI3K-AKT signaling pathway (like *PIK3CA*, *PTEN*, *MTOR*) (Pappas et al., 1993; Moirangthem et al., 2021). It is noteworthy that some rare overgrowth syndromes remain molecularly uncharacterized. Mehawej et al. (2023) recently reported a Lebanese boy with Moreno-Nishimura-Schmidt overgrowth syndrome without a genetic mutation to delineate the phenotype. Further epigenetic studies revealed a different methylation status of several CpG sites between this patient and healthy controls (Mehawej et al., 2023). Significant genetic heterogeneity of similar neurodevelopmental disorders increases the difficulty in clinical diagnosis and promotes the application of high-throughput sequencing technology (Wilfert et al., 2017; Sun et al., 2022). In this article, we identity a novel pathogenic *SETD2* variant (c.5835_c.5836insAGAA, p. A1946Rfs*2) in a Chinese 3-year-old boy without overgrowth, expanding the phenotype and genotype spectrum of *SETD2* mutation. The proband received a 20-month rehabilitation training for movement, cognition, speech and sense in the rehabilitation center. The rehabilitation training was effective but the progress was slow. The boy could only speak simple words and could not jump by the time of submitting manuscript, showing mild motor and speech development delay. As the grown-up patients demonstrated various degrees of developmental delay, long term follow-up is necessary for the boy to get accurate phenotype.

*SETD2*, located at chromosome3p21.31, has two coding transcripts (NM_014159.7 and NM_001349370.3). NM_014159.7 is the longer isoform, containing 21 exons and encoding the SETD2 protein with 2564aa. The wild SETD2 protein is evolutionally conserved and is composed of AWS (associated with SET domains) domain (1495–1549aa), SET (Su(var)3-9, Enhancer-of-zeste, Trithorax) domain (1550–1673aa), PostSET domain (1674–1690aa), SHI domain (2164–2213 aa), WW domain (2391–2422aa) and SRI domain (2469–2548aa) (Bhattacharya et al., 2021). The SET domain mediates methylation of histone H3-lysine 36 residue, tubulin and STAT1 (Hacker et al., 2016); the SHI domain is responsible for alternative splicing by interacting with hnRNP L (Bhattacharya et al., 2021); the WW domain have been identified to interact with the proline-rich region huntingtin protein (Gao et al., 2014); the SRI domain interacts the largest subunit of RNA polymerase II (Rpb1); the SRI domain directs SETD2 activity toward genes that are actively transcribed (Li et al., 2005). The SETD2 protein reveals a critical and conserved role in epigenetic and transcriptional regulation.

Xu and his colleagues have demonstrated that SETD2 is required for proper cortical arealization and the formation of cortico-thalamo-cortical circuits (Xu et al., 2021), suggesting the importance of SETD2 in neurological development. *SETD2* conditional knockout mice exhibit defects in social interaction, motor learning, and spatial memory, reminiscent of patients with the Sotos-like syndrome bearing *SETD2* mutations. According to the review of *SETD2* mutations, most patients show speech and language developmental delay, motor developmental delay, variable degree of intellectual disability and behavioral problems, which is completely in accord with the symptoms of *SETD2* knockout mice. Another feature of Luscan-Lumish syndrome is overgrowth. Height and weight may normalize in adulthood, but macrocephaly is usually present at all ages (Pappas et al., 1993). Since only the latest records of growth parameters are collected, the rate of high stature or obesity is obvious lower than that of macrocephaly. Advanced bone age could be regarded as a reflect of bone overgrowth.

Frameshift mutations and non-sense mutations account for 68.5% (35/51) of *SETD2* point mutations, suggesting that Luscan-Lumish syndrome is likely due to loss of function of SETD2. But we failed to find an association between genotype and phenotype of *SETD2* mutations, which suggest a hypothesis that all the *SETD2* point mutations will lead to the decreased activity of methyltransferase and result in H3K36me3 loss. Taking the novel mutation c.5835_c.5836insAGAA p.(A1946Rfs*2) we identified, for example, it would generate a truncated SETD2 protein losing the conserved domains in the C-terminal region. The mutated transcripts harboring premature termination codons (PTCs) is also probably degraded by the non-sense-mediated mRNA decay (NMD). Duns et al. (2010) found that the expression of *SETD2* transcripts was upregulated treated with inhibitors of NMD in the renal cell carcinoma cell lines RCC-ER and RCC-AB, which harbor hemizygous mutations introducing PTCs in *SETD2*. This finding suggests that the degradation of the truncated SETD2 protein by the NMD is possible.

In this article, the case demonstrates motor and speech development delay early in life. He has cognitive impairment and will probably develop in to moderate intellectual disability in the future, as the most patients with Luscan-Lumish syndrome do. He has no overgrowth, which is a major feature of Luscan-Lumish syndrome. He has no facial and cranial deformities. He has no epilepsy nor multisystem malformations. He has experienced recurrent otitis and pneumonia, which has been reported in some affected children with Luscan-Lumish syndrome (Luscan et al., 2014; van Rij et al., 2018). As only four patients with *SETD2* mutations experienced recurrent otitis and the onset age was younger than 5 years old, no solid evidences support that Luscan-Lumish syndrome is associated with immune deficiency or another disease is co-occurring in the patient we reported. This case expands the mutation spectrum and phenotype spectrum of *SETD2* mutations. Moreover, the patients with heterozygous missense mutation *SETD2* c.5218C>T p.(R1740W) exhibit profound intellectual disability, microcephaly, congenital anomalies affecting several organ systems (Rabin et al., 2020), which are significantly different from the symptoms of Luscan-Lumish syndrome and are diagnosed as Rabin-Pappas syndrome (RAPAS, MIM#620155). The patients with heterozygous missense mutation *SETD2* c.5219G>A p.(R1740Q) lack major medical malformations and present normal growth (Rabin et al., 2020), which are diagnosed as Intellectual developmental disorder 70 (MRD70, MIM#620157). The intellectual disability of MRD70 patients is more serious than that of patients with Luscan-Lumish syndrome, but not as severe as that of RAPAS patients (Rabin et al., 2020). These findings indicate that codon 1740 appears to be critically important for normal *SETD2* function and the mutations in codon 1740 may induce abnormal nervous development *via* an alternative mechanism.

In addition to point mutations of nuclear genes, CNVs and mtDNA variants are also responsible for part of neurodevelopmental disorders (Lam et al., 2019; Ullah et al., 2021; Silva et al., 2022; Sun et al., 2022). D’Gama has reported a case (AN00090) harboring a germline missense mutations in *SETD2* predicted to be deleterious (responsible for ASD) and a 15q deletion consistent with her diagnosis of Angelman syndrome (D'Gama et al., 2015). Thus CNVdetection and mtDNA sequencing have been applied for the genetic diagnosis of the case in this article. No suspicious variants have been found by CNV detecion and mtDNA sequencing, which further suggests the pathogenicity of *SETD2*c.5835_c.5836insAGAA (p.A1946Rfs*2) in the proband.

In conclusion, we have identified a novel pathogenic *SETD2* variant in a Chinese 3-year-old Chinese boy with global development delay and without overgrowth. Our findings expand the genotype-phenotype knowledge of *SETD2-*associated neurological disorder and provide new evidence for further genetic counselling.

## Data Availability

According to national legislation/guidelines, specifically the Administrative Regulations of the People’s Republic of China on Human Genetic Resources (http://www.gov.cn/zhengce/content/2019-06/10/content_5398829.htm, http://english.www.gov.cn/policies/latest_releases/2019/06/10/content_281476708945462.htm), no additional raw data is available at this time. Data of this project can be accessed after an approval application to the China National Genebank (CNGB, https://db.cngb.org/cnsa/). Please refer to https://db.cngb.org/, or email: CNGBdb@cngb.org for detailed application guidance. The accession code CNP0003966 should be included in the application.
